# Printability, Mechanical Response, and Surface Integrity of MEX-Manufactured Gyroid Lattices with Uniform and Graded Cell Sizes

**DOI:** 10.3390/polym18131664

**Published:** 2026-07-04

**Authors:** Ray Tahir Mushtaq, Ghulam Hassan Askari, Mudassar Rehman, Rakan Albarakati, Yanen Wang, Aqib Mashood Khan

**Affiliations:** 1 Institute for Interdisciplinary Innovation Research, Xi’an University of Architecture and Technology, Xi’an 710055, China; 2 Bio-Additive Manufacturing University-Enterprise Joint Research Center of Shaanxi Province, Department of Industry Engineering, Northwestern Polytechnical University, Xi’an 710072, China; engrghulamhassanaskari@mail.nwpu.edu.cn; 3 Interdisciplinary Research Center for Intelligent Manufacturing & Robotics (IRC IMR), King Fahd University of Petroleum & Minerals (KFUPM), Dhahran 31261, Saudi Arabia; mudassar.rehman@kfupm.edu.sa; 4 Industrial Engineering Department, College of Engineering and Architecture, Umm Al-Qura University, Makkah 21955, Saudi Arabia; rmbarakati@uqu.edu.sa; 5 College of Mechanical and Electrical Engineering, Nanjing University of Aeronautics and Astronautics, Nanjing 210016, China; dr.aqib@nuaa.edu.cn

**Keywords:** gyroid lattice, material extrusion, printability map, graded cell size, compressive response, surface roughness, energy absorption

## Abstract

Triply periodic minimal surface (TPMS) gyroid lattices are promising lightweight and energy-absorbing polymer structures, but their manufacturability by material extrusion (MEX) depends strongly on cell size, grading direction, and relative density. This study investigates PLA gyroid lattices with uniform and graded cell-size configurations using initial and final cell sizes of 1, 1.5, and 2 mm and target relative densities of 10, 20, and 30%. A full-factorial design was used to construct a printability map, followed by quasi-static compression testing, areal surface-roughness characterization, and SEM observation of representative specimens. The printability results showed that low-density fine-cell configurations were most prone to incomplete wall formation and collapse, whereas the 30% relative-density group was printable for all investigated cell-size combinations. Under compression, the 30% relative-density uniform 1 mm gyroid showed the highest maximum stress among the tested configurations, while graded structures terminating in smaller cells also provided favorable load bearing and energy-absorption behavior. The plateau stability index, calculated from stress fluctuations between collapse and densification, helped distinguish stable progressive collapse from more oscillatory deformation. Surface roughness and SEM observations further indicated that smoother, more continuous wall surfaces were associated with more uniform deformation, whereas rougher and defect-rich surfaces promoted localized buckling, cracking, and brittle collapse. Overall, the results identify experimentally supported relationships between gyroid cell-size configuration, printability, surface integrity, and compressive response within the investigated PLA MEX design space.

## 1. Introduction

Additive manufacturing (AM) has enabled the fabrication of complex lattice and cellular structures with superior strength-to-weight ratios, controllable porosity, and high design freedom for applications in aerospace, automotive, biomedical implants, and impact-absorbing components. Material extrusion (MEX), as a thermoplastic extrusion-based AM process, is especially attractive for polymer lattices because it is economical, allows rapid prototyping, and is compatible with widely used biocompatible materials such as PLA. However, MEX parts inherently exhibit relatively low geometric resolution, anisotropic layer-by-layer bonding, and staircase-induced surface roughness, which become particularly critical when printing thin-walled lattice structures with small cell sizes [1].

Within this context, triply periodic minimal surface (TPMS) lattices—especially gyroid geometries—have received significant attention because of their smooth, continuous profiles, high specific surface area, and efficient load-bearing behaviour. Polymer TPMS gyroid structures fabricated by MEX have recently been explored as lightweight structural elements, energy absorbers, and bone-mimicking scaffolds, owing to their ability to combine low density with relatively high stiffness and tailored deformation modes. Earlier work on cellular solids has established relative density as a primary predictor of effective modulus, strength, and energy absorption, and this trend remains valid for AM lattices, where relative density (or volumetric porosity) is often regarded as the single most important structural characteristic for mechanical performance. Consequently, many studies have focused on how unit cell geometry and relative density together determine the mechanical response of gyroid and other TPMS lattices produced by additive processes [2].

Netto et al. [1] investigated the compressive behaviour of MEX-fabricated PLA gyroid structures and systematically varied cell size and wall thickness to quantify their effects on elastic modulus, plateau stability, and failure modes at different relative densities. Their results showed that smaller cell sizes and thicker walls can enhance stiffness and plateau stress but also increase defect sensitivity and the likelihood of local buckling in thin features. Musthafa et al. [3] performed in silico prediction of the mechanical behaviour of uniform gyroid scaffolds for bone tissue engineering and demonstrated that changes in unit cell size and wall thickness at fixed porosity significantly influence the effective stiffness and stress distribution in the scaffold. These works highlight that geometric parameters such as cell size and wall thickness cannot be chosen independently of relative density when designing high-performance polymer gyroid lattices.

Beyond uniform designs, graded and non-uniform gyroid structures have been proposed to exploit spatial variations in geometry for improved energy absorption and tailored stiffness. Alemayehu and Todoh [4] experimentally investigated bioinspired composite TPMS gyroid lattices fabricated via MEX and demonstrated enhanced energy absorption characteristics compared to conventional designs. Similarly, Jones et al. [5] studied additively manufactured multimaterial gyroid lattices and showed that increasing unit cell size (geometric scale) reduces mechanical performance due to fewer cells per unit volume and increased boundary-dominated effects.

The influence of cell size on mechanical stability has also been investigated in more generic cellular materials. Gong et al. [6] numerically studied microcellular polypropylene and reported that larger cell sizes lead to reduced mechanical performance due to unfavorable stress states, whereas smaller and more uniform cells enhance mechanical properties. In additively manufactured TPMS structures, graded geometry has likewise been shown to influence compressive response and energy absorption. Yu et al. [7] designed and fabricated uniform- and graded-density Schwarz P and Gyroid TPMS structures and showed that grading altered deformation characteristics and energy absorption behavior. Furthermore, manufacturing-induced imperfections such as porosity and surface roughness have been shown to alter stress distributions and degrade lattice performance [8]. In addition, in situ monitoring methods have highlighted the importance of detecting defects during fabrication to improve structural quality and reliability [9].

To provide context for the present study, Table 1 summarizes key experimental and numerical studies on MEX-fabricated polymer TPMS and gyroid lattices, highlighting their investigated geometries, relative densities, mechanical performance, and consideration of printability and surface quality. The comparison reveals that existing studies typically focus on either limited cell size ranges or isolated performance metrics, with limited attention to the combined effects of grading, printability, and surface morphology.

Recent DOE-based studies on PLA MEX have demonstrated that print quality and mechanical response depend on coupled processing and design variables rather than on single parameters alone. Maalihan et al. [10] applied a multi-response central composite design and desirability-function approach to optimize PLA MEX processing, while Auffray et al. [11] used a Taguchi/DOE approach to evaluate the effects of layer height, infill density, print velocity, raster orientation, outline overlap, and extrusion temperature on PLA tensile properties. In contrast to process-parameter studies on standard PLA specimens, the present work focuses on geometry-driven manufacturability and mechanical response in explicitly modelled gyroid lattices by varying initial cell size, final cell size, and relative density in a full factorial design.

The material state of PLA can also influence MEX repeatability because thermal history, storage conditions, and ageing may affect filament behavior and printed-part properties [12]. Recent work on PLA filament ageing after MEX processing has shown that post-processing history and ageing conditions should be considered when interpreting polymer AM results. In the present work, the same Creality PLA filament type and the same printing parameter set were used for all configurations so that the observed differences could be primarily associated with cell-size configuration and relative density.

**Table 1:** Comparative Analysis of Studies on MEX-Fabricated Polymer Gyroid Lattices.

Study	Material & Design	Cell Size/Density	Mechanical Performance	Characterization & Printability
Netto et al. [1]	PLA, MEX gyroids	2–5 mm, ~15–25%	Yield, plateau, failure vs. size & thickness	Limited morphology; no quantitative roughness; qualitative printability
Musthafa et al. [3]	PLA gyroids (simulation)	Uniform, varied porosity	Predicted stiffness & strength (scaffold design)	No experimental validation; printability not considered
Riot et al. [13]	AM polymer lattices	Variable relative densities with geometrical imperfections	Energy absorption sensitivity to defects and density	Defect modeling and analysis; no printability framework
Present Study	PLA, MEX gyroids (uniform + graded)	1–2 mm, 10–30%	Full compressive response + energy absorption + stability index	Quantitative roughness (Sa, Sq, Sz, etc.), SEM, and DOE-based printability map

Despite this literature on TPMS lattices, energy-absorbing structures, and graded architectures, the combined roles of initial cell size, final cell size, and relative density in MEX-manufactured PLA gyroid lattices remain insufficiently clarified. Existing studies generally focus on a limited range of uniform cell sizes, process parameters, or isolated mechanical indicators, whereas thin-walled gyroid lattices require simultaneous consideration of printability, geometric configuration, compressive response, and surface integrity.

To address these gaps, the present study performs a full-factorial investigation of MEX-printed PLA gyroid lattices with systematically varied initial cell size, final cell size, and nominal relative density. Uniform and graded cell-size configurations of 1, 1.5, and 2 mm were evaluated at 10, 20, and 30% relative density. The work combines printability mapping, quasi-static compression, plateau-stability analysis, energy absorption calculation, confocal roughness characterization, and SEM observation before and after compression. This combined approach enables the relationships among manufacturability, lattice architecture, surface quality, and compressive failure behavior to be evaluated in a single experimental framework.

## 2. Experiments: Materials and Methods

### 2.1. Materials

Commercial Creality PLA filament (Shenzhen Creality 3D Technology Co., Ltd., Shenzhen, China) with a nominal diameter of 1.75 mm was used to fabricate all lattice specimens by MEX. PLA was selected because of its good printability, widespread use in additively manufactured polymer lattice structures, and suitability for lightweight structural and energy-absorbing applications. The filament was stored under dry conditions before printing and was used without an additional drying step.

### 2.2. Gyroid Design and CAD Generation

All specimens were designed as sheet-based triply periodic minimal surface (TPMS) gyroid lattices. The gyroid geometry was generated using MSLattice software from an implicit level-set representation and was periodically repeated in three dimensions to form cubic lattice blocks. The terms initial cell size and final cell size refer to the unit-cell dimensions at the beginning and end of the build direction, respectively. For uniform lattices, the initial and final cell sizes were identical. For graded lattices, the cell size was varied monotonically along the build direction to produce a through-thickness gradient.

Three nominal cell sizes were investigated: 1, 1.5, and 2 mm. These values were combined to generate uniform configurations of 1→1, 1.5→1.5, and 2→2 mm, as well as graded configurations of 1→1.5, 1→2, 1.5→1, 1.5→2, 2→1, and 2→1.5 mm. The arrow notation indicates the change in unit-cell size from the beginning to the end of the build direction. Three target relative densities were considered: 10%, 20%, and 30%.

### 2.3. Experimental Matrix

**Figure 1:**
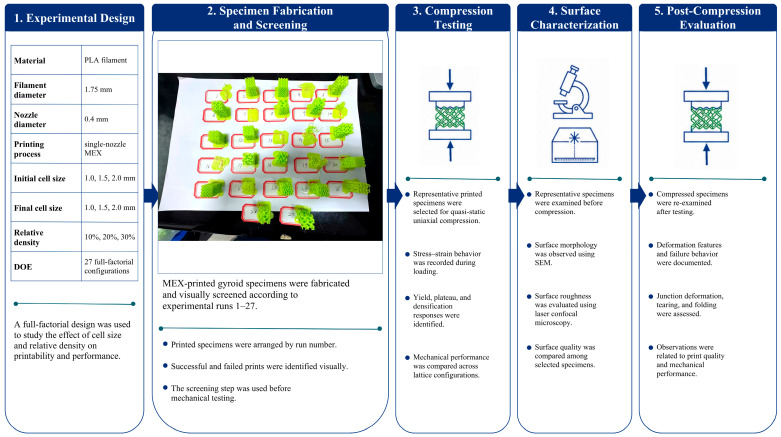
Materials and methods workflow for MEX-printed PLA gyroid lattice fabrication and evaluation.

A full factorial design of experiments was used to evaluate the combined effects of initial cell size, final cell size, and relative density on printability and compressive response (see Table 2, Figure 1). Each factor was investigated at three levels, producing a 3 × 3 × 3 matrix with 27 unique gyroid configurations. Each configuration was assigned an experiment number according to the experimental matrix used in Section 3.

At least three specimens from each configuration were fabricated for printability assessment. Configurations that were successfully printed were subsequently used for compression testing. The successfully printed configurations were Experiments 2, 3, 5, 6, 8, 9, 11, 12, 15, 17, 18, 20, 21, 23, 24, 25, 26, and 27. The unprinted configurations were excluded from mechanical testing because their intended geometry could not be retained during fabrication.

The 30% relative-density group was used as the main equal-density comparison set because all nine cell-size combinations were successfully printed at this density as summarized in Table 3. This allowed the influence of cell-size configuration to be examined without the confounding effect of print failure.

### 2.4. Material Extrusion and Printability Assessment

The specimens were fabricated using a Creality K1C MEX printer (Shenzhen Creality 3D Technology Co., Ltd., Shenzhen, China) equipped with a 0.4 mm nozzle and a heated build plate. CAD models were exported as STL files and processed using Creality Print. Because the gyroid architecture was explicitly generated in MSLattice, the specimens were sliced and printed as fully modelled lattice structures rather than using a slicer-generated gyroid infill. The same printing conditions (see Table 4) were maintained for all configurations to isolate the effects of cell size and relative density as taken from [14]. The lattice blocks were oriented such that the build direction coincided with the Z-axis of the gyroid structure. For graded specimens, the cell-size gradient was also aligned with this direction. Internal support structures were not used because the continuous gyroid sheet architecture was self-supporting during layer-by-layer deposition.

Printability was assessed using predefined acceptance criteria. A specimen was classified as successfully printed when the lattice retained its intended gyroid geometry, continuous gyroid walls were formed, the structure remained dimensionally stable during printing, and the specimen could be removed from the build plate without collapse or severe damage. A specimen was classified as failed when severe warping, incomplete wall formation, poor interlayer bonding, wall collapse, or loss of lattice integrity was observed. The resulting printability response was recorded as a binary outcome for each of the 27 DOE configurations and used to construct the printability map, where 1 denotes a successfully printed configuration and 0 denotes a failed configuration.

### 2.5. Compression Testing and Mechanical Data Reduction

Quasi-static compression testing was conducted on the 3D-printed PLA gyroid lattice samples measuring 15 mm × 15 mm × 20 mm using a GTM 2500 instrument (GTM Testing and Metrology GmbH, Bickenbach, Germany). The specimens were compressed between two parallel platens under displacement control at a crosshead speed of 1.3 mm/min. Testing was performed at a controlled temperature of 25 °C. Each specimen was compressed up to 60% engineering strain to determine the compressive strength, elastic modulus, deformation response, plateau behavior, densification behavior, and energy absorption. These tests were used to evaluate the structural integrity and load-bearing capability of the printed PLA gyroid lattices under different cell-size configurations and relative-density levels.

Engineering stress and engineering strain were calculated using the original cross-sectional area and initial height of each specimen. All compression tests were performed along the build direction (Z-axis). For graded lattices, the cell-size gradient was aligned with the loading direction, meaning that the initial and final cell-size regions were loaded in series through the specimen thickness. The specimens were tested in the as-built orientation, with the build-start face placed on the lower platen and the build-end face contacted by the upper platen.

The resulting engineering stress–strain curves were used to extract the elastic modulus, yield stress, collapse stress, densification stress, maximum compressive stress, plateau stability index, and energy absorption. The elastic modulus was obtained from the slope of the initial linear region of the stress–strain curve. Yield stress was determined using the 0.2% offset method. Collapse stress was identified as the stress associated with the first major deviation from the initial elastic response and the onset of progressive lattice collapse. Densification stress was taken as the stress at the beginning of the rapid stress increase caused by cell-wall contact and compaction. The highest stress reached within the tested strain range was reported as the maximum compressive stress. The term ultimate tensile stress was not used because all mechanical tests were performed under compression.

### 2.6. Plateau Stability and Energy Absorption

The plateau stability index was calculated from the stress fluctuation within the plateau interval. In this study, the plateau interval was defined objectively as the strain region between the collapse point and the onset of densification for each stress–strain curve. The mean plateau stress (μp) and standard deviation of plateau stress (σp) were calculated within this interval, and the stability index was defined in Equation (1) as [15],

SI = 1 − (σp/μp)(1)

A value closer to 1 indicates a smoother and more stable plateau, whereas a lower value indicates stronger stress oscillation during progressive collapse. The index was interpreted together with maximum stress and energy absorption because a high SI alone does not necessarily indicate the best load-bearing or energy-absorbing performance. Energy absorption was calculated from the area under the engineering stress–strain curve up to 50% strain, as shown in Equation (2) [15].

(2) $$W_{0.5}=\int_{0}^{0.5}\sigma (\varepsilon )d\varepsilon$$

In Equation (2), W_0.5_ is the volumetric energy absorption up to 50% engineering strain and σ(ε) is the engineering stress as a function of engineering strain. The integration was performed numerically using the trapezoidal rule applied directly to the experimental stress–strain data. When stress is expressed in MPa, the calculated energy absorption is reported in MJ/m^3^.

### 2.7. Surface Roughness Characterization

Surface roughness measurements were conducted using confocal scanning microscopy to obtain high-resolution surface topography of the printed gyroid walls. A Sensofar (Barcelona, Spain) optical profilometer equipped with a 20× lens was used. The scanned area was 877.20 × 660 μm with a pixel size of 1.29 μm/pixel. The Z-scan range was 109 μm. The Coarse Shift algorithm was used with a threshold of 5.00%. The measurement light intensity was maintained at 4.69%, and no ring light was applied (0.00%). Each scan required 16 s and achieved a measurement coverage of 25.92%. For each selected lattice configuration, three different measurement zones on external gyroid wall regions were analyzed.

Areal surface texture parameters were calculated according to ISO 25178 [16] surface-metrology practice. ISO 25178-compliant filtering [16] was applied using an S-filter with a short-wavelength cut-off of 8.00 μm and an L-filter with a long-wavelength cut-off of 250.00 μm. These filters were used to remove high-frequency noise and large-scale waviness before parameter extraction. Polynomial leveling of degree 3 was applied for surface leveling, missing data points were restored using the bicubic method, and outliers were removed using a mean-threshold criterion. The arithmetical mean height (Sa), root mean square height (Sq), and maximum surface height (Sz) were used to quantify the roughness magnitude, while skewness (Ssk) and kurtosis (Sku) described the height-distribution characteristics.

### 2.8. Scanning Electron Microscopy

Scanning electron microscopy was performed using a COXEM (Daejeon, Republic of Korea) SEM system to examine surface morphology, printing-induced defects, and deformation mechanisms before and after compression. Before SEM observation, the specimens were gently cleaned with an air blower to remove loose debris. Because PLA is electrically insulating, the specimens were coated with a thin gold layer by sputtering to improve surface conductivity and image quality.

Three representative specimens were selected for SEM characterization: Specimen 3, corresponding to the uniform fine-cell 1→1 mm lattice; Specimen 12, corresponding to the graded 1.5→1 mm lattice; and Specimen 25, corresponding to a coarse-cell 2→2 mm gyroid lattice selected from the printable design space. This selection enabled comparison among a fine uniform lattice, a graded lattice terminating in a fine cell size, and a coarse-cell gyroid configuration from the printable design space.

Pre-compression SEM images were used to evaluate wall morphology, wall continuity, layer bonding, surface defects, pores, voids, and junction quality. Post-compression SEM images were acquired from fractured or highly deformed regions to identify the dominant failure mechanisms, including local wall buckling, crack initiation and propagation, interlayer delamination, material tearing, and collapse at nodal junctions. The SEM observations were interpreted together with surface roughness data and compression-test results to support the discussion of manufacturing quality, surface morphology, and compressive failure behaviour in the gyroid lattices.

## 3. Results and Discussion

### 3.1. Printability Challenge

The printability map shows a strong combined effect of relative density and cell-size configuration. At 10% relative density, only the coarse 2→2 mm configuration was successfully printed, corresponding to a configuration-level success rate of 1/9 within this density group. At 20% relative density, 8 of 9 configurations were successfully printed, while at 30% relative density all 9 cell-size combinations were printable. Overall, 18 of the 27 DOE configurations were successfully fabricated. These results indicate that low-density fine-cell gyroids are most vulnerable to unstable wall formation, whereas increasing relative density improves wall continuity, structural self-support, and printing reliability.

#### 3.1.1. Cell Size and Printing Issues

Small-cell configurations were more sensitive to printing instability because reduced wall thickness and short deposition paths increased the likelihood of discontinuous material deposition, local distortion, and insufficient wall support during layer formation. Coarser 2 mm cell-size configurations provided larger unsupported feature lengths but also thicker and more stable walls, which improved the ability of the structure to retain its geometry during fabrication.

#### 3.1.2. Relative Density and Printing Status:

**Figure 2:**
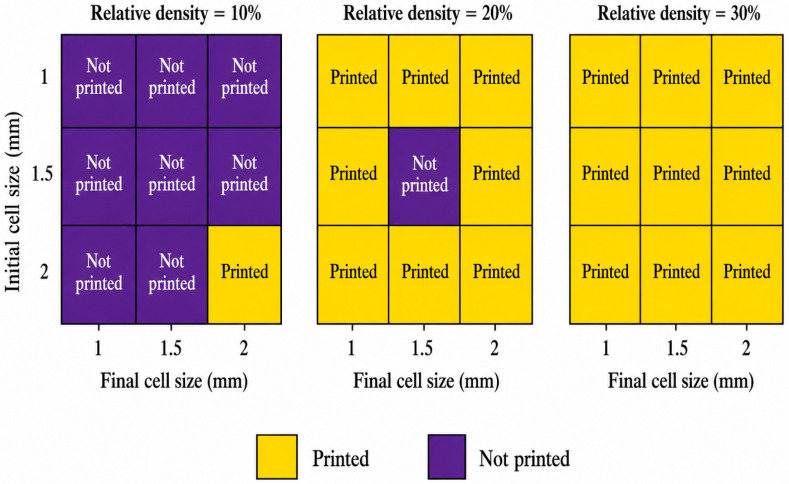
Two-dimensional printability maps of MEX-printed PLA gyroid lattices as functions of initial cell size, final cell size, and relative density.

Structures with low relative densities (around 10%) were generally unstable during printing. The combination of minimal material volume and thin lattice walls often led to warping or incomplete layers. In contrast, structures with medium to high relative densities (20–30%) had more material per unit volume, providing enough stability for successful printing. This trend was clear, as prints with 20% or 30% densities were more likely to succeed compared to those with 10%. The overall printability was influenced by the balance between cell size and relative density. Smaller cell sizes and lower densities resulted in higher failure rates, while larger cell sizes and higher densities improved printing success. However, at extremely high densities, issues with material flow and over-extrusion could arise. As shown in Figure 2, printability depended on the combined effect of initial cell size, final cell size, and relative density rather than on any single factor.

Figure 2 presents three two-dimensional printability maps corresponding to 10%, 20%, and 30% relative density. In each panel, the horizontal axis represents final cell size and the vertical axis represents initial cell size. Yellow cells indicate successful prints and purple cells indicate failed builds. This representation directly shows how printability changes with the combined effects of initial cell size, final cell size, and relative density.

### 3.2. Compressive Response Analysis

**Figure 3:**
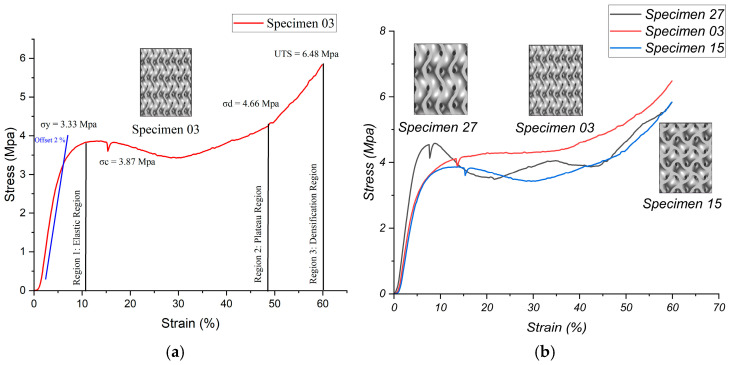
Compressive stress–strain curves of 30% relative-density gyroid lattices: (**a**) labelled response regions of Specimen 3; (**b**) comparison of uniform cell sizes, Specimens 3, 15, and 27.

The compression tests provided valuable insights into the mechanical performance of gyroid lattice structures with different cell size configurations, all maintaining a consistent relative density of 30%. The stress–strain curve for Specimen 3 highlights the unique mechanical response of a gyroid lattice under compressive loading, showing its elastic, plateau, and densification regions (as shown in Figure 3a). This linear relationship indicates the material’s stiffness, which is reflected in its elastic modulus and its ability to resist deformation. The yield stress (σ_y_ = 3.33 MPa) marks the point where the material transitions from elastic to plastic behavior, identified using the 0.2% offset method. After this, the material begins to experience irreversible deformation.

Once the elastic region ends, the structure enters the plateau region, where stress levels off even as strain continues to increase. This phase is key for energy absorption, demonstrating the gyroid’s ability to deform while efficiently distributing the load. The collapse stress (σ_c_ = 3.87 MPa) marks the point at which plastic deformation starts in the lattice struts, signifying the beginning of this region. In Specimen 3, the plateau region remains comparatively stable, with minimal waviness in the stress–strain curve. This stability is important for ensuring consistent performance under extended loading, making the gyroid ideal for impact absorption applications.

As the plateau phase continues, the structure enters the densification region, which is marked by the densification stress (σ_d_ = 4.66 MPa). In this phase, the voids within the lattice collapse completely, and the stress increases sharply as the material resists further compression. The maximum compressive stress (σ_max_ = 6.48 MPa) represents the peak stress right before failure, showcasing the structure’s strength. The performance of each configuration is then summarized and compared based on key factors such as maximum stress, elastic modulus, yield strength, and plateau stability. Below, the discussion is broken down into two categories: lattices with the same initial and final cell sizes, and those with different initial and final cell sizes.

#### 3.2.1. Impact of Unit Cell Size on Mechanical Strength

##### Impact of Identical Initial and Final Cell Sizes

Smaller uniform cell sizes (Specimen 3) showed better mechanical properties, including the highest maximum stress and the most stable plateau phase, as noted by Netto et al. [1]. Larger uniform cell sizes (Specimens 15 and 27) faced issues with reduced material efficiency and higher stress concentrations [3] as shown in Figure 3b. The evaluation of structures where the initial and final unit cell sizes are the same sheds light on how uniform cell sizes affect mechanical performance. Specimen 3, with an initial and final cell size of 1 mm, achieved a maximum stress of 6.48 MPa. The stress–strain curve demonstrates its high maximum stress and excellent plateau stability. The smaller, uniform cell size creates a dense network of load-distributing struts, which improves stress resistance and material efficiency [17]. The extended plateau and significant stress increase at high strain further highlight its superior energy absorption capacity [4]. Specimen 3 stands out as the best-performing configuration, offering the highest stress tolerance thanks to its fine, uniform lattice structure.

Specimen 15, with an initial and final cell size of 1.5 mm, reached a maximum stress of 5.83 MPa. The stress–strain curve for this uniform 1.5 mm cell size shows moderate load-bearing capacity but lacks the complex stress distribution seen in finer lattices. The plateau phase exhibits slight oscillations, suggesting reduced stability under compression. The smaller number of struts in the lattice limits material efficiency compared to finer configurations. While Specimen 15 performs reasonably well, it is outperformed by finer configurations like Specimen 3. Similarly, Specimen 27, with a 2 mm initial and final cell size, also reached a maximum stress of 5.83 MPa. The stress–strain curve for this 2 mm cell size shows the lowest stiffness and the most unstable plateau phase among the uniform configurations. Stress oscillations are more pronounced, indicating poor load distribution and significant stress concentration. The larger cell size reduces both material efficiency and structural stability. Specimen 27 performs similarly to Specimen 15 but is weaker in terms of stability and efficiency due to its larger cell size. Jones et al. [5] explored the impact of larger cell sizes on mechanical strength, noting that larger unit cells result in fewer cells per unit volume. This can lead to less uniform material distribution and increased susceptibility to localized stress concentrations, ultimately reducing overall strength.

Specimen 3 outperformed all the others, achieving the highest maximum stress, the steepest elastic slope, and the most stable plateau. Smaller, uniform cell sizes proved more effective at distributing stress and maintaining stability, as demonstrated by Specimen 3, in contrast to the larger cell sizes of Specimens 15 and 27.

##### Impact on Differing Initial and Final Cell Sizes

**Figure 4:**
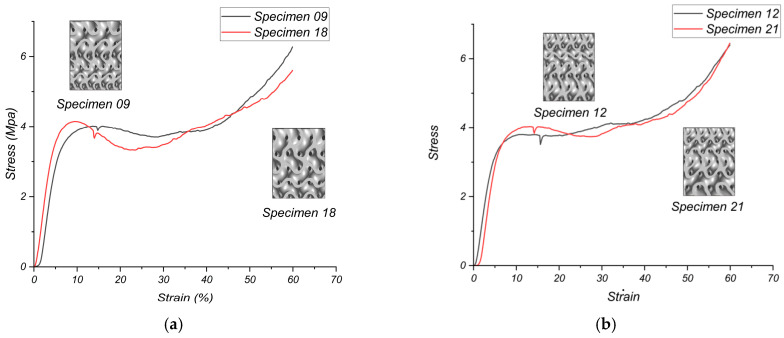
Compressive stress–strain response of graded gyroid lattices at 30% relative density: (**a**) increasing cell-size gradients ending in coarse cells, Specimen 9 (1→2 mm) and Specimen 18 (1.5→2 mm); (**b**) decreasing cell-size gradients ending in fine cells, Specimen 12 (1.5→1 mm) and Specimen 21 (2→1 mm).

Transitioning to larger final cell sizes (Specimens 9 and 18) introduced stress instability and reduced maximum stress, highlighting the downsides of coarser lattice configurations. Specimen 9, with a 1 mm initial and 2 mm final cell size, reached a maximum stress of 6.27 MPa. The stress–strain curve for this transition, from a smaller initial cell size to a larger final cell size, creates a balance between finer and coarser lattice features. This approach aligns with Dai et al.’s research on non-uniform gyroid structures, where a gradient from small to large cells shows improved mechanical energy absorption. This improvement is due to the layer-by-layer collapsing mechanism, which offers a more controlled deformation process under stress [17]. The plateau phase shows slight oscillations, and the maximum stress is slightly lower than Specimen 3, reflecting reduced material efficiency from the larger final cell size. While it performs well, the shift to a larger final cell size limits overall efficiency. Similarly, Specimen 18, with a 1.5 mm initial and 2 mm final cell size, reached a maximum stress of 5.6 MPa. The stress–strain curve for this transition shows reduced load distribution efficiency and significant plateau instability. Stress oscillations are more pronounced, indicating poor stress resistance [18]. Specimen 18 performs the weakest among the transition configurations due to the disadvantages of the larger final cell size. The effect of gradient direction is shown in Figure 4a,b, where gradients ending in finer cells preserve higher load-bearing capacity than gradients ending in coarser cells.

Transitions to smaller final cell sizes (Specimens 12 and 21) improved stress resistance and plateau stability, resulting in mechanical performance that is comparable to or slightly below that of Specimen 3. Specimen 12, with a 1.5 mm initial and 1 mm final cell size, reached a maximum stress of 6.41 MPa. The stress–strain curve for this reverse transition to a smaller final cell size improves stress distribution, leading to a stable plateau and high maximum stress, as noted in the research by Dawei Li et al. Starting with larger cell sizes at the top can create a stiffer structure with a higher elastic modulus, especially in sheet-based gyroid structures. This configuration can be advantageous for applications that require greater stiffness [19]. The stress–strain curve for Specimen 12 closely mirrors that of Specimen 3, demonstrating the benefits of a finer final cell size. Specimen 12 stands out as one of the best-performing configurations, achieving mechanical properties similar to Specimen 3. Similarly, Specimen 21, with a 2 mm initial and 1 mm final cell size, reached a maximum stress of 6.44 MPa, which is very close to Specimen 3 and higher than Specimen 12. The curve behavior for this reverse transition also demonstrates exceptional load distribution and stress resistance, with a stable plateau phase. This further underscore the importance of a finer final cell size. Specimen 21 ranks among the top-performing configurations, yielding results nearly identical to those of Specimen 3.

**Figure 5:**
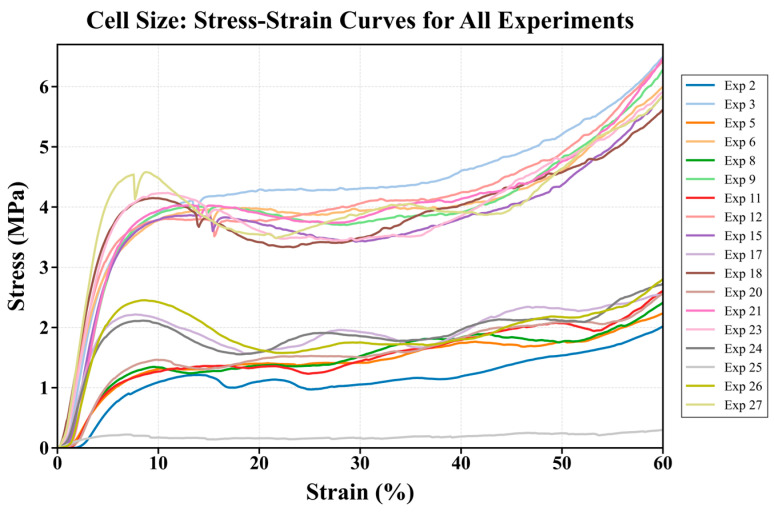
Stress–strain curves of all successfully printed gyroid lattice configurations, showing the effects of cell-size grading and relative density on compressive response.

Specimen 21 closely matches Specimen 3 in both maximum stress and plateau stability, demonstrating that transitioning to a finer final cell size (1 mm) effectively boosts mechanical performance. Specimen 12 also performs well, benefiting from a similar transition to a smaller final cell size. In contrast, configurations with larger final cell sizes (Specimens 9 and 18) performed worse, highlighting the negative impact of coarser lattice structures on stress distribution and stability. The complete comparison in Figure 5 confirms that relative density controls the overall stress level, whereas the cell-size pathway controls plateau waviness and densification behavior.

Specimen 3, with its uniform 1 mm cell size, remains the standout performer due to its finely distributed lattice structure. Specimens 21 (transitioning from 2 mm to 1 mm) and 12 (transitioning from 1.5 mm to 1 mm) follow closely, showcasing the benefits of transitioning to a smaller final cell size. Smaller cell sizes, whether uniform or at the final stage of a transition, significantly enhance mechanical performance by improving stress distribution, material efficiency, and plateau stability. Larger cell sizes reduce these advantages, highlighting the importance of optimizing lattice geometry.

#### 3.2.2. Impact of Unit Cell Size on Stability Index

**Figure 6:**
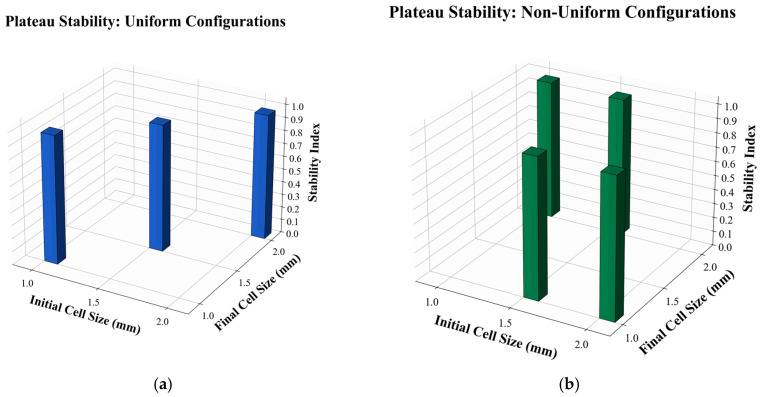
Plateau stability index of gyroid lattices at 30% relative density: (**a**) uniform cell-size configurations; (**b**) graded cell-size configurations.

The plateau stability index was used to compare stress fluctuations during progressive collapse of the 30% relative-density gyroid lattices. As shown in Figure 6, the uniform fine-cell lattice, Specimen 3, exhibited a high stability index, indicating a comparatively smooth and regular plateau response. The medium and coarse uniform lattices showed lower stability, which is consistent with stronger stress oscillations caused by less uniform load transfer and localized deformation. Among graded lattices, configurations ending in finer cells, particularly Specimens 12 and 21, maintained relatively stable plateau responses, whereas gradients ending in coarser cells showed more pronounced fluctuations. These results indicate that smaller uniform cells or finer final-cell regions can improve plateau regularity by promoting more distributed collapse and reducing abrupt local buckling events.

Figure 6a,b compares the plateau stability index for uniform and graded configurations at 30% relative density. The uniform fine-cell lattice, Specimen 3, showed a high stability index and a regular plateau response, while the medium and coarse uniform lattices showed stronger stress oscillations. Among graded lattices, the stability response depended on both the gradient direction and the complete stress–strain behavior. Although isolated SI values may appear close among different configurations, the combined interpretation of SI, maximum stress, energy absorption, and deformation mode indicates that fine uniform and selected fine-ended graded lattices provided the most favorable overall collapse stability. Therefore, the stability index was not used as an isolated ranking parameter but as one descriptor of plateau regularity.

#### 3.2.3. Impact of Unit Cell Size on Energy Absorption

Energy absorption was evaluated up to 50% engineering strain and interpreted together with plateau stress and densification behavior. The 30% relative-density group showed that the uniform fine-cell lattice, Specimen 3, achieved the highest energy absorption among the selected configurations. Graded specimens ending in smaller cells, particularly Specimens 12 and 21, retained moderate-to-high energy absorption because the finer final-cell region promoted more distributed collapse. In contrast, configurations ending in coarser cells showed more stress oscillation and less efficient energy absorption [20]. The energy-absorption results show that high plateau stress and delayed densification both contribute to improved absorption capacity. Therefore, the energy-absorption response should be interpreted together with maximum stress and plateau stability [21,22].

##### Impact of Uniform Initial and Final Cell Sizes

Specimen 3 exhibited the highest energy absorption among the uniform configurations (1.58 MJ/m^3^), which is consistent with its higher stress level across the plateau and densification regions. The uniform fine-cell lattice provides a dense network of stress-distributing pathways and delays localized failure, thereby improving energy absorption [23]. Specimen 15, with a uniform 1.5 mm cell size, showed the lowest energy absorption (1.38 MJ/m^3^), mainly because the coarser lattice provides fewer load paths and lower plateau stress than the fine-cell configuration [24]. Specimen 27, with a uniform 2 mm cell size, showed moderate-to-high energy absorption (1.52 MJ/m^3^), but its larger cell size produced fewer load-bearing pathways and more pronounced stress fluctuations [13,25]. Therefore, Specimen 3 provided the most favorable energy-absorption response among the uniform lattices, followed by Specimen 27 and Specimen 15.

##### Impact of Differing Initial and Final Cell Sizes

**Figure 7:**
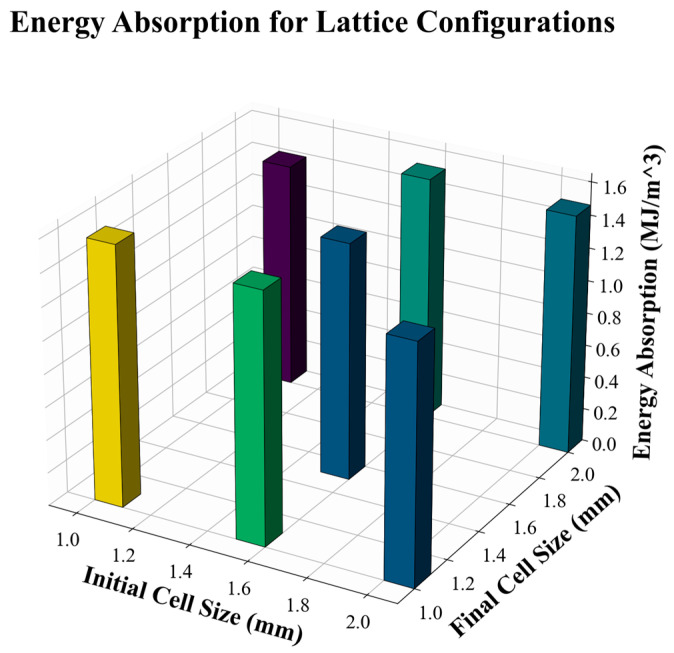
Energy absorption of selected gyroid lattice configurations up to 50% compressive strain.

In specimen 9, with a unit cell size from 1 mm to 2 mm, the energy absorption of 1.44 MJ/m^3^ is moderate, with stress levels declining slightly during the plateau phase due to the transition to a larger final cell size. The larger final cell size reduces the density of struts in the lattice, limiting its ability to maintain high stress across the plateau [26]. Whereas specimen 12 ranging unit cell size 1.5 mm to 1 mm, the energy absorption of 1.49 MJ/m^3^ is improved. The reverse transition to a smaller final cell size enhances stress levels and stability during the plateau. The smaller final cell size maximizes load-bearing capacity and energy absorption by increasing the density of stress pathways [27]. The energy-absorption comparison in Figure 7 and Table 5 shows that Specimen 3 provides the highest W_0.5, while reverse-gradient specimens maintain moderate-to-high energy absorption but do not exceed the fine uniform design.

Specimen 18, with a 1.5→2 mm cell-size transition, showed an energy absorption of 1.44 MJ/m^3^, which was similar to that of Specimen 9. This response can be attributed to the coarser final cell size, which reduced material efficiency, increased stress concentration, and produced a less stable plateau response [28]. Specimen 21, with a 2→1 mm transition, achieved moderate-to-high energy absorption (1.46 MJ/m^3^) because the finer final-cell region helped maintain load transfer during plateau deformation. However, this value did not exceed the uniform fine-cell Specimen 3. Overall, Specimens 12 and 21 performed best among the decreasing-gradient configurations, whereas Specimens 9 and 18 showed lower energy-absorption efficiency because their gradients terminated in coarser cells. These results confirm that a finer final-cell region can improve stress distribution and energy absorption, while the uniform 1 mm configuration remains the most effective design within the tested 30% relative-density group.

### 3.3. Surface Roughness Analysis

**Figure 8:**
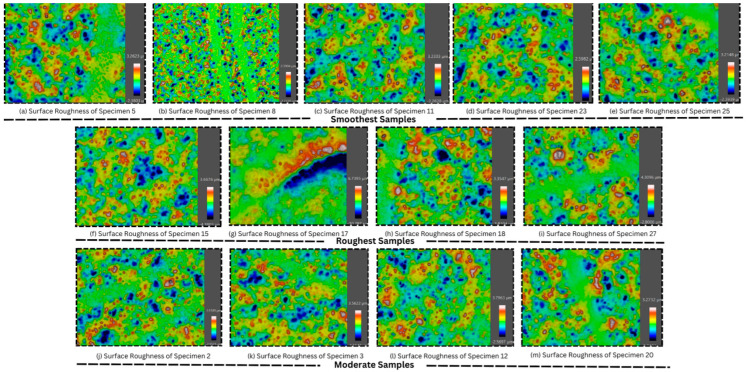
Confocal surface-height maps of selected MEX-printed PLA gyroid lattices grouped according to surface-roughness condition. (**a**–**e**) Lowest-roughness specimens: Specimens 5, 8, 11, 23, and 25; (**f**–**i**) highest-roughness specimens: Specimens 15, 17, 18, and 27; (**j**–**m**) intermediate-roughness specimens: Specimens 2, 3, 12, and 20.

Surface roughness was evaluated using confocal microscopy to examine the association between manufacturing quality, surface integrity, and compressive response. Figure 8 shows representative height maps grouped by surface condition. The roughness data reveal a wide range of surface states, from very smooth profiles with low Sa and Sz values to highly irregular surfaces containing sharp peaks and deep valleys. These height variations are relevant because local peaks and valleys can act as stress raisers during compression, particularly at gyroid wall junctions and curved wall segments [7].

In Specimen 3, surface roughness increased compared to Specimen 2, as indicated by a Sa of 0.4274 µm and a total height variation (Sz) of 6.9612 µm. The root means square height (Sq) of 0.5679 µm shows a larger contribution of peaks and valleys. Peaks reached a height of 3.5843 µm (Sp), and valleys dipped to −3.3769 µm (Sv), confirming increased irregularities. A kurtosis (Sku) value of 4.396 suggests a moderately peaked surface, with a skewness (Ssk) of 0.07, indicating a slight predominance of peaks. The confocal image supports this with broader red zones (peaks) and blue regions (valleys), reflecting more distributed roughness. The roughness maps in Figure 8 show that surface quality varies strongly among printable designs, with localized peaks and valleys even when average Sa values are similar.

Specimen 12 exhibited slightly increased roughness compared to Specimen 11, with a Sa of 0.387 µm and a total height variation (Sz) of 6.3661 µm. Peaks reached 3.7845 µm, and valleys dipped to −2.5816 µm, increasing variability. The kurtosis (Sku) of 6.1336 highlights sharp peaks, while the skewness (Ssk) of 0.5039 indicates a predominance of peaks over valleys. The confocal microscopy image shows a mixture of yellow-green zones, red peaks, and deeper blue valleys. The surface roughness of Specimen 15 increased notably, with a Sa of 0.4556 µm and a total height variation (Sz) of 8.0479 µm. Peaks reached 3.6778 µm, and valleys extended to −4.3701 µm, highlighting deeper irregularities. The kurtosis (Sku) value of 4.5599 and skewness (Ssk) of −0.1519 reveal a slight dominance of valleys. The confocal image supports this, with widespread blue zones (valleys) and scattered red peaks contributing to roughness. The surface of Specimen 18 displayed moderate roughness, with a Sa of 0.4568 µm and an Sz of 7.1651 µm. Peaks reached 3.3087 µm, and valleys dipped to −3.8565 µm, showing noticeable variability. The kurtosis (Sku) of 5.0179 and skewness (Ssk) of −0.1404 indicate a slight dominance of valleys. The confocal image confirms moderate roughness, with a balance of yellow-green zones, red peaks, and blue valleys. Specimen 27 displayed roughness with a Sa of 0.399 µm and an Sz of 7.1102 µm. Peaks reached 4.3118 µm, and valleys dipped to −2.7984 µm. The kurtosis (Sku) of 9.5536 and skewness (Ssk) of 1.1734 reveal a predominance of sharp peaks. The confocal image confirms this with yellow-green zones and elevated red peaks.

The surface-roughness results were interpreted together with the compression curves and SEM observations. Specimens with lower Sa and Sq generally showed more continuous deposited roads and fewer severe surface defects, which corresponded to smoother progressive deformation under compression. In contrast, specimens with higher Sz and visible local defects exhibited more pronounced stress oscillations, localized buckling, and crack initiation at rough or poorly bonded regions. Therefore, the roughness data support a mechanistic relationship between surface integrity and compressive failure mode. However, this interpretation is restricted to the tested representative specimens and is not presented as a universal statistical correlation for all MEX gyroid lattices.

#### Comparative Analysis

The roughness results can be classified into three groups. The smoothest specimens were Experiments 8, 23, 5, 11, and 25, which exhibited lower Sa and Sz values and more consistent height maps. Experiment 8 was the smoothest surface overall (Sa = 0.2361 µm and Sz = 4.6714 µm), indicating stable extrusion and limited peak-valley variation. Experiments 23 and 25 also showed controlled height distributions, suggesting that a coarse-cell geometry does not automatically produce a rough surface if the local deposition path remains stable.

The roughest group included Experiments 17, 18, 27, and 15. Experiment 17 was a clear outlier, with Sa = 0.8460 µm, Sq = 1.3925 µm, and Sz = 17.037 µm, indicating extreme height variation. Such roughness can originate from local over-extrusion, insufficient interlayer bonding, thermal shrinkage, or unstable deposition around curved TPMS wall segments. Experiments 15, 18, and 27 also showed elevated Sz values, confirming that large peaks or deep valleys remained on the printed wall surfaces. These features are mechanically important because they increase local notch effects and can accelerate crack initiation during compression.

The moderate group, including Experiments 2, 3, 12, and 20, showed balanced roughness with localized but not extreme peaks and valleys. This group is especially important because Specimens 3 and 12 also exhibited high compressive performance. Their response indicates that moderate roughness does not necessarily prevent stable collapse when the lattice architecture provides sufficient load-distributing pathways. Conversely, roughness becomes more damaging when combined with coarse cells or junction defects, as fewer load paths are available to redistribute stress around local imperfections.

**Figure 9:**
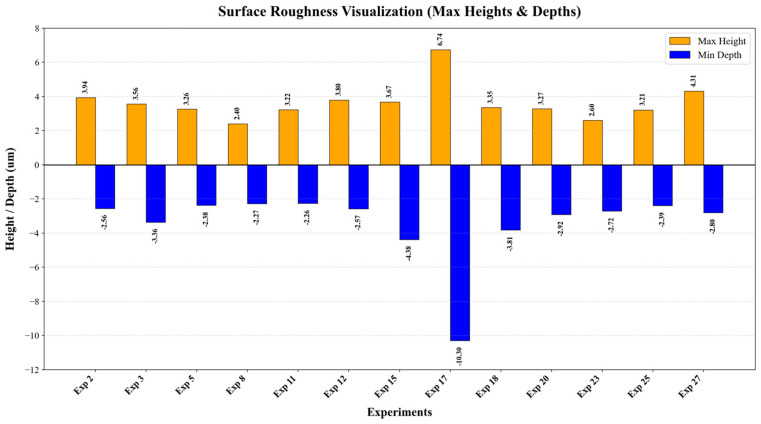
Comparison of maximum peak height and maximum valley depth for selected gyroid lattice surfaces.

These observations agree with previous researchers who reported that AM surface roughness, porosity, and geometric defects can perturb local stress fields and reduce lattice reliability [7]. They also support the cell-size findings of Netto et al. [1] and Gong et al. [6], where fine and more uniform cellular architectures provided more stable mechanical responses. In the present gyroids, the best mechanical response therefore results from the combined effect of topology and surface integrity: fine or fine-ended cell configurations supply more load paths, while smoother surfaces reduce defect-driven crack initiation. Figure 9 highlights the peak-valley contribution to this behaviour, and the quantitative roughness data are listed in Table 6.

### 3.4. SEM Analysis

SEM was used to examine the surface morphology, local defects, and deformation features of representative gyroid lattice structures before and after compression. Three representative specimens were compared: Specimen 3, corresponding to the uniform fine-cell 1→1 mm lattice; Specimen 12, corresponding to the graded 1.5→1 mm lattice; and Specimen 25, corresponding to a coarse-cell 2→2 mm gyroid lattice selected from the printable design space. These specimens were selected to compare fine uniform, graded, and coarse-cell gyroid configurations.

#### 3.4.1. SEM Analysis Before Compression

**Figure 10:**
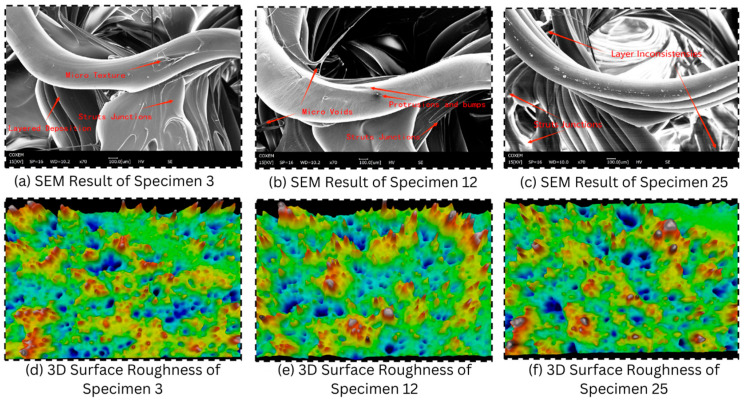
Pre-compression SEM micrographs and three-dimensional surface-height maps of selected MEX-printed PLA gyroid lattices. (**a**–**c**) SEM micrographs of Specimens 3, 12, and 25, respectively, showing gyroid-wall morphology, strut junctions, layer-deposition traces, micro-texture, micro-voids, protrusions, and local layer inconsistencies; (**d**–**f**) corresponding three-dimensional surface-roughness maps of Specimens 3, 12, and 25, respectively, showing differences in surface-height distribution before compression.

Before compression, the SEM images showed clear differences in wall morphology, layer continuity, and local defect formation among the selected specimens. Specimen 3 exhibited relatively continuous gyroid walls and well-defined junctions, indicating stable deposition in the fine uniform lattice. Although Specimen 3 was not the smoothest surface among all measured specimens, its continuous fine-cell architecture provided a dense network of load-distributing pathways (see Figure 10).

Specimen 12 showed moderate surface irregularities and local imperfections near gyroid wall junctions. These features are consistent with the graded 1.5→1 mm configuration, where the transition in cell size can modify local deposition paths and wall continuity. The observed imperfections may act as local stress-concentration sites during compression, but the finer final-cell region still provides additional load-distributing pathways compared with coarser configurations.

Specimen 25 showed coarse wall morphology and visible localized defects. Its average Sa value was not the highest among all measured surfaces; therefore, the discussion emphasizes localized defect morphology and higher Sz-related peak–valley variation rather than describing it as the roughest surface by Sa. The coarser morphology and fewer local load-distributing pathways may increase sensitivity to defect-driven deformation and crack initiation during compression.

#### 3.4.2. SEM Analysis After Compression

After compression, the SEM images revealed different deformation and failure features among the selected gyroid configurations. Specimen 3 showed comparatively distributed deformation, with localized tearing and limited crack propagation along the gyroid walls. The fine uniform architecture helped redistribute stress through multiple load paths, which is consistent with its high maximum compressive stress and stable plateau response.

Specimen 12 exhibited localized buckling, wall tearing, and crack initiation near junctions and transition regions. These features suggest that the graded geometry influenced the deformation path by concentrating local strain in specific wall regions. However, because the configuration terminated in a finer 1 mm cell region, the lattice still maintained relatively favorable load-bearing behavior compared with configurations ending in coarser cells.

Specimen 25 showed more pronounced localized collapse, material folding, and visible wall damage after compression. The coarse-cell morphology provided fewer local load-distributing pathways than the fine uniform lattice, making the deformation more sensitive to local wall defects and peak–valley surface features. These observations support the interpretation that surface integrity and cell-size configuration jointly influence compressive failure behavior.

#### 3.4.3. Relationship Between Surface Morphology and Compression Response

**Figure 11:**
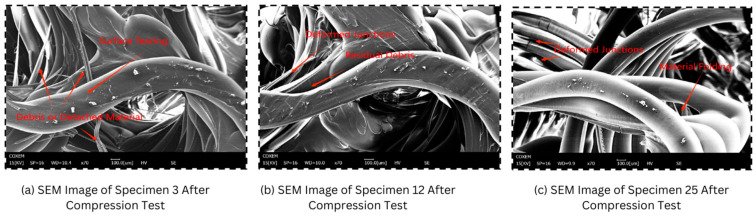
Post-compression SEM micrographs of selected MEX-printed PLA gyroid lattices after quasi-static compression testing. (**a**) Specimen 3 showing surface tearing and detached debris on the deformed gyroid wall; (**b**) Specimen 12 showing deformed strut junctions and residual debris after compression; (**c**) Specimen 25 showing junction deformation and material folding.

The SEM observations were interpreted together with the surface-roughness data and compression results as seen in Figure 11. Specimen 3 demonstrated that a fine and continuous gyroid wall network can support distributed deformation even when moderate roughness is present. Specimen 12 showed that graded lattices ending in a finer cell size can maintain favorable mechanical response, although local defects and junction irregularities may promote localized cracking. Specimen 25 showed that coarse-cell morphology and localized surface defects can contribute to stronger deformation localization and post-compression damage. Therefore, the SEM results support a mechanistic relationship among cell-size configuration, surface integrity, and compressive failure mode, but this interpretation is limited to the selected representative specimens and is not presented as a universal statistical correlation.

## 4. Conclusions

This study investigated the printability, compressive response, surface integrity, and failure behavior of MEX-printed PLA gyroid lattices with uniform and graded cell sizes. The DOE-based printability map showed that manufacturability depended on the combined effect of initial cell size, final cell size, and relative density. Low-density fine-cell gyroids were most susceptible to wall instability and print failure, whereas all investigated cell-size combinations were printable at 30% relative density. Compression testing showed that the uniform 1 mm gyroid achieved the highest maximum stress among the 30% relative-density specimens, while graded configurations terminating in smaller cells also showed favorable energy absorption and progressive collapse behavior. The plateau stability index provided a useful measure of stress fluctuation in the collapse plateau, but it was interpreted together with maximum stress, energy absorption, and densification behavior. Surface roughness and SEM observations indicated that smoother and more continuous printed walls supported more uniform deformation, whereas rougher and defect-rich surfaces were associated with local buckling, cracking, and unstable collapse in the representative specimens. These results provide experimentally supported guidance for selecting cell-size configuration and relative density in PLA MEX gyroid lattices designed for lightweight and energy-absorbing applications within the investigated design space.

## Figures and Tables

**Table 2:** Experimental factors used in the gyroid lattice design.

Factor	Levels	Description
Initial cell size	1, 1.5, 2 mm	Unit-cell size at the beginning of the build direction
Final cell size	1, 1.5, 2 mm	Unit-cell size at the end of the build direction
Relative density	10%, 20%, 30%	Nominal solid volume fraction of the lattice

**Table 3:** Thirty percent relative-density configurations used for equal-density comparison.

Specimen	Initial Cell Size (mm)	Final Cell Size (mm)	Configuration Type
3	1	1	Uniform fine cell
6	1	1.5	Graded, increasing cell size
9	1	2	Graded, increasing cell size
12	1.5	1	Graded, decreasing cell size
15	1.5	1.5	Uniform medium cell
18	1.5	2	Graded, increasing cell size
21	2	1	Graded, decreasing cell size
24	2	1.5	Graded, decreasing cell size
27	2	2	Uniform coarse cell

**Table 4:** Printing conditions used for all MEX gyroid lattice specimens.

Print Parameter	Value
Printer	Creality K1C
Printing process	MEX
Slicer	Creality Print
Filament	Creality PLA, 1.75 mm
Nozzle diameter	0.4 mm
Nozzle temperature	200 °C
Build-plate temperature	60 °C
Layer thickness	0.2 mm
Print speed	60 mm/s
Cooling fan	100%
Support structures	Not used

**Table 5:** Energy Absorption Performance of Uniform and Transition Lattice Configurations.

Configuration	Cell Size	Energy Absorption MJ/m^3^ (Quantitative)	Energy Absorption (Qualitative)	Key Reason
Specimen 3	Uniform (1 mm)	1.58	Very High	Finer lattice maximizes stress pathways and plateau stability.
Specimen 15	Uniform (1.5 mm)	1.38	Lowest	Coarser lattice reduces load distribution efficiency.
Specimen 27	Uniform (2 mm)	1.52	High	Significant stress oscillations and fewer stress pathways.
Specimen 9	Transition (1 mm→2 mm)	1.44	Moderate	Larger final cell size reduces stress levels in the plateau phase.
Specimen 12	Transition (1.5 mm→1 mm)	1.49	Moderate	Reverse transition enhances stress distribution and stability.
Specimen 18	Transition (1.5 mm→2 mm)	1.44	Moderate	Coarser final cell size reduces material efficiency and stability.
Specimen 21	Transition (2 mm→1 mm)	1.46	Moderate	Reverse transition maintains high stress levels and enhances energy absorption.

**Table 6:** Surface roughness parameters and qualitative observations for selected printed gyroid lattices.

Runs	Sa (µm)	Sq (µm)	Sz (µm)	Observations
Exp 2	0.3414	0.4797	6.4964	Moderately smooth, minimal peaks
Exp 3	0.4274	0.5679	6.9612	Increased roughness, balanced
Exp 5	0.3919	0.5294	5.7238	Moderate roughness, consistent
Exp 8	0.2361	0.3600	4.6714	Smoothest surface
Exp 11	0.3425	0.4556	5.4931	Moderately smooth, minimal peaks
Exp 12	0.3870	0.5242	6.3661	Slightly rougher, localized peaks
Exp 15	0.4556	0.6031	8.0479	Rough surface, deeper valleys
Exp 17	0.8460	1.3925	17.037	Roughest surface, extreme values
Exp 18	0.4568	0.6128	7.1651	Moderate roughness, balanced
Exp 20	0.4258	0.5742	6.1913	Moderate roughness, balanced
Exp 23	0.4137	0.5427	5.3137	Smooth, consistent surface
Exp 25	0.3861	0.5451	5.6087	Moderately smooth, controlled
Exp 27	0.3990	0.5760	7.1102	Rougher surface, sharp peaks

## Data Availability

The data that supports the findings of this study are available within the manuscript.
